# Low rate of recurrence of *Helicobacter Pylori* infection in spite of high clarithromycin resistance in Pakistan

**DOI:** 10.1186/1471-230X-13-33

**Published:** 2013-02-21

**Authors:** Javed Yakoob, Shahab Abid, Wasim Jafri, Zaigham Abbas, Khalid Mumtaz, Saeed Hamid, Rashida Ahmed

**Affiliations:** 1 Department of Medicine, Aga Khan University, Stadium Road, Karachi, 74800, Pakistan; 2 Department of Pathology, Aga Khan University, Stadium Road, Karachi, 74800, Pakistan

**Keywords:** *Helicobacter pylori*, Clarithromycin resistance, Recurrence, Nonulcer dyspepsia

## Abstract

**Background:**

The aim was to investigate the reinfection rate of *H. pylori* during a follow-up period of 12 months in adults who had undergone eradication therapy.

**Methods:**

One hundred-twenty patients; 116 with gastritis, 3 with duodenal ulcer and 1 gastric ulcer, were studied. Their mean age was 41 ± 13 years (range 18–77) and male: female ratio of 2:1. *H. pylori* were cultured and antibiotic sensitivity was determined by Epsilometer test (E-test) for clarithromycin (CLR) and amoxicillin (AMX). Primers of *urease C* gene of *H. pylori* and Sau-3 and Hha I restriction enzymes were used for polymerase chain reaction-restriction fragment length polymorphism analysis (PCR-RFLP). ^14^C urea breath test (^14^C-UBT) was performed 4 weeks after the eradication therapy. The successfully treated patients were observed for 12 months with ^14^C-UBT to assess *H. pylori* status. If ^14^C-UBT was negative, it was repeated after every 12 weeks. If ^14^C-UBT was positive, endoscopy was repeated with biopsies.

**Result:**

The eradication therapy was successful in 102(85%) patients. Out of forty-seven *H. pylori* isolates cultured, clarithromycin sensitivity was present in 30(64%) and amoxicillin in 45(98%), respectively. Follow-up ^14^C-urea breath tests of all 102 patients who eradicated *H. pylori* remained negative up to 9 months. However, in 6 patients, the ^14^C-UBT confirmed recurrence at 12 months. The recurrence rate was 6%.

**Conclusion:**

A low rate of recurrence of *H. pylori* infection was found in patients with dyspeptic symptoms. *H. pylori* isolates demonstrated a high invitro clarithromycin resistance.

## Background

*Helicobacter pylori* (*H. pylori*) is a Gram negative microorganism that has been categorized as a class I carcinogen [1]. It is associated with gastritis, gastric and duodenal ulcers, gastric adenocarcinoma, and mucosa-associated lymphoid tissue lymphoma [2]. Long term chronic gastritis associated with *H. pylori* is known to progress to glandular atrophy and intestinal metaplasia (IM) over a period of 12 years [3]. *Helicobacter pylori* gastritis may lead to multifocal atrophy and IM, gradually expanding from the antrum to the body [3]. A long term study with five year follow up from China have reported no change in the degree of intestinal metaplasia (IM) and atrophy after successful eradication [3]. In other long term randomized controlled trials there was a significant regression of preneoplastic lesions such as atrophy and IM among those who had cleared the infection after five to six years of follow up [4,5]. A definite cure of peptic ulcer disease and prevention of ulcer complications, as well as cure of mucosa-associated lymphoid tissue (MALT) lymphoma is dependent on successful eradication of *H. pylori*.

Recurrence of *H. pylori* after a successful eradication is rare in developed countries and more frequent in developing countries [6]. Recrudescence is recolonization of the same strain while reinfection is colonization with a new strain. Reinfection is considered to be more likely to be responsible for most of the cases [6]. This differentiation is difficult and requires utilization of molecular fingerprinting techniques to confirm that the identified bacteria, before and after therapy, are genetically identical [7]. Recrudescence results from treatment failure while reinfection is considered a problem of heavy *H. pylori* contamination of the environment, drinking water, institutionalized patients, medical personnel or family members, especially in developing countries [6]. In developing countries, *H. pylori* infection is widespread.

Regional studies available from Bangladesh, India and Iran have shown varied recurrence rates [8-10]. In a recent study in Karachi in apparently healthy children, *H. pylori* seroprevalence in children aged 11–15 years was 54% [11]. *H. pylori* seropositivity increased with age and in low-middle socioeconomic status [11]. However, local studies addressing the issue of recurrence of *H. pylori* are not available. In this study we investigated the recurrence rate of *H. pylori* during a follow-up period of 12 months in adults who had undergone eradication therapy.

## Methods

### Patients

One hundred-twenty consecutive patients with dyspepsia attending the endoscopy suite of gastroenterology section of Aga Khan University Hospital were enrolled from April 2008–June 2010. There were eighty males and forty females (age range 17–80 years, mean age 41 ± 13 years; Table 1). An informed consent was taken from all patients and the Aga Khan University ethics review committee approved the study. Only those who were willing to comply with the follow-up schedule and gave informed consent were enrolled in the study. Pregnant and lactating women, patients with esophageal or gastric tumors and patients with liver cirrhosis and esophageal varices were excluded. In addition, those who had received antisecretory drugs, antibiotics, nonsteroidal anti-inflammatory drugs, corticosteroids or bismuth-containing drugs during the preceding 8 weeks were also excluded. All the patients were native residents of the city. Endoscopic examination was conducted using Olympus video endoscopes (Olympus Tokyo, Japan), and the presence of lesions in the esophageal and gastroduodenal mucosa was noted. Gastric biopsy specimens were obtained three each from the antrum and corpus for rapid urease test (RUT), histology, culture and PCR for *H. pylori* urease C gene. The biopsies from the antrum were obtained 2–3 cm from the pylorus, and from the body, midway between the antral-body junction and the cardia. Patients who were positive for *H. pylori* infection by means of ^14^C-UBT with RUT or histology received treatment comprising of a proton pump inhibitor (esomeprazole) 20 mg BD (twice a day) for 10 days and clarithromycin 500 mg BD and amoxicillin 500 mg BD for 7 days. Two weeks after completion of treatment, ^14^C-UBT was performed to document eradication of *H. pylori* infection. Patients with positive ^14^C-UBT were excluded from the study while those with negative were followed up every 3 months by a ^14^C-UBT for 12 months until it tested positive and was followed by endoscopy and biopsy.

**Table 1:** Patients characteristics

**Age (years)**	
Mean ± SD	41 ± 13
Range	18-77
**Gender**	
Male	80(67)*
Female	40(33)
**Diagnosis**	
Gastritis	116(97)
Gastric ulcer	1(1)
Duodenal ulcer	3(2)
**Histology**	
Grade 1	61(51)
Grade 2	54(45)
Grade 3	5(4)
**Antibiotic susceptibility pattern**	**n = 47**
Clarithromycin	
Sensitive	30(64)
Resistance	17(36)
Amoxicillin	
Sensitive	45(96)
Resistance	2(4)
***H. pylori *DNA fingerprint (PCR-RFLP)**	
Same on both sides	102(85)
Different on both sides	18(15)

*n (%) = number and percentage.

### Sample size

The required sample size is 103 individuals, calculated on the basis of an estimated prevalence of reinfection of 13% and bound on error of estimation specified to be at the most .065 (6.5%) and a confidence level of 95% [8].

### Rapid urease test

The tissue specimens were used for the RUT (Pronto dry, Medical Instrument Corp, France) consisting of a dry filter paper enriched with urea, phenol red (a pH indicator), buffers and a bacteriostatic agent in a sealed plastic slide [12]. *H. pylori* urease enzyme present in the biopsy tissue sample decomposed urea to cause a pH rise that changed color of the dot from yellow to a bright magenta. Pronto Dry results were read in 30 minutes and one hour. The color change from yellow to pink was considered a positive result and no color change as negative for Pronto Dry.

### Histological analysis

Formalin-fixed and paraffin-embedded gastric biopsy specimens were routinely processed. Gastritis activity was graded on a four-point scale of none (grade 0), mild (grade 1), moderate (grade 2), and severe (grade 3) according to the guidelines of the Sydney system [13]. The presence of *H. pylori* was assessed on modified Giemsa-stained sections.

### ^14^C- *Urea breath test*

Patients swallowed 37 kBq (1 μCi) of an encapsulated form of 14C-urea/citric acid composition (Helicap, Noster System AB, Sweden) with water after endoscopy [14]. Breath samples were collected with a special dry cartridge system (Heliprobe Breath Card, Noster System AB, Sweden) after 10 min. Patients exhaled gently into the cartridge mouthpiece until the indicator membrane changed in color from orange to yellow. Breath card was inserted into a β-scintillation counter (Heliprobe-analyser, Noster System AB Stockholm, Sweden) and activity was counted for 250 s. Results were expressed both as counts per minute (HCPM) and as grade (0: not infected, CPM < 25; 1: equivocal, CPM 25–50; 2: infected, CPM > 50), which was suggested by the manufacturer according to the counts obtained from the cartridges. Grades 0 and 1 were considered negative for the detection of *H. pylori*. In a previous study the accuracy of ^14^C-UBT was compared to histology and RUT. Accuracy of ^14^C-UBT was 93% in comparison with histology while its positive and negative predictive values were 97% and 84%, respectively [14]. Comparison of ^14^C-UBT with RUT gives an accuracy of 96%, with positive and negative predictive values of 95% and 97%, respectively [14].

### Culture and identification of H. pylori

The specimens were transported immediately in sterile phosphate buffered saline to isolate *H. pylori.* Thus, within three hours of collection each specimen was homogenized and the resulting suspension was inoculated onto Columbia Blood Agar (Oxoid) medium and Dents supplement (containing vancomycin, trimethoprim and polymyxin) and incubated at 37°C under microaerophilic conditions for 4–7 days. Plates were then examined for bacterial growth and typical colonies were selected for identification. The identity of *H. pylori* was confirmed by Gram stain, urease and catalase test. One half of the homogenate was used for culture, and the other half was kept at −80°C for future DNA extraction. *H. pylori* isolates were defined as gram-negative spiral-shaped bacilli that were catalase positive and rapidly (less than 1 h) urease positive. *H. pylori* NCTC 11637 (type strain) was used as a positive control for the culture conditions and identification tests.

### Antibiotic susceptibility testing

Antibiotic susceptibility was determined on Mueller Hinton agar (Oxoid, UK) containing 10% defibrinated sheep blood and a cell suspension calibrated at 3 McFarland units by the Epsilometer (E-test) using clarithromycin and amoxicillin E-test strips (AB Biodisk, Solna, Sweden). Plates were read after 3 days of incubation at 37°C. The tests were carried out according to the manufacturer’s instructions. *H. pylori* NCTC 11637 was used as a sensitive control.

### Extraction of genomic DNA

DNA was extracted from gastric tissue as described before [15]. Samples were stored at −20°C before PCR amplification was performed. DNA content and purity was determined by measuring the absorbance at 260 nm and 280 nm using a spectrophotometer (Beckman DU-600, USA).

### PCR amplification for H. pylori ure C gene

PCR was performed using extracted DNA as the template and urease gene C for primers. Forward primer (5’-TGGGACTGATGGCGTGAGGG-3’) and reverse primer (5’-AAGGGCGTTTTTAGATTTTT-3’) were prepared from the urease gene sequence according to the report of Labigne et al. [16]. PCR amplification was carried out in a total volume of 50 μL containing 2 μL of 2 mmol/L dNTPs, 1 μL containing 50 ρmol of primer 1, 1 μL containing 50 ρmol of primer 2 (synthesized by ABI Automatic synthesizer), 1 unit of Taq DNA polymerase (Promega), 5 μL of 10 × PCR reaction buffer, 3 mmol/L of MgCl_2_, 2 μL of DNA template containing 0.5 ng of extracted DNA and total volume rounded to 50 μL by double distilled water. The reaction was carried out in a Perkin Elmer 9700 thermal cycler. The amplification cycle consisted of an initial denaturation of target DNA at 95°C for 5 min and then denaturation at 94°C for 1 min, primer annealing at 56°C for 1 min and extension at 72°C for 1 min. The final cycle included an extension step for 5 min at 72°C to ensure full extension of the product. Samples were amplified through 35 consecutive cycles. Negative reagent control reactions were performed with each batch of amplifications, consisting of tubes containing distilled water in place of the DNA samples. Five μL of PCR product was electrophoresed on a 1.5% agarose gel to ensure homogeneity and yield. PCR amplification resulted in a homogeneous DNA fragment of the expected size of 820 bp for ure C gene.

### PCR-RFLP

The amplified products obtained by PCR were subjected to restriction endonuclease digestion for 2 hours at 37°C in 20 microliter (μl) volume, as recommended [17]. The digested samples were analyzed by agarose gel (3%, wt/volume) electrophoresis. Restriction enzyme Sau-3 (5U) and Hha I (5 U), (New England Biolabs) were used on the basis of sequence data available for this amplified product. The restriction enzyme HhaI recognized on the 820-bp *UreC* gene amplified PCR product restriction site 5’…GCG^↓^ C …3’ giving 2 fragments varying in size from 100 bps to 550 bps and showing 4 band patterns while restriction enzyme Sau3A1 recognized site 5’…^↓^ GATC..3’giving 3 fragments ranging in size from 50 bps to 600 bps having 7 bands patterns, respectively. RFLP analysis by HhaI and Sau 3A together generated eleven distinguishable digestion patterns. Small variations (<10 bps) in the size of the restriction fragments were not considered a different pattern. In case of an identical restriction pattern from antrum and body, the patient was considered to have an infection by the same *H. pylori* strain while by restriction fragments that exceeded amplified fragment size and yielded different restriction fragments, patient was considered to be infected by different *H. pylori* strains.

### Statistical assessment

The statistical package for social science SPSS (Release 16, standard version, copyright © SPSS; 1989–2007) was used for data analysis. The descriptive analysis was done for demographic and clinical features. Results were presented as mean ± standard deviation for quantitative variables and number (percentage) for qualitative variables. Differences in proportion were assessed by using Pearson Chi square, Fisher exact or likelihood ratio test where appropriate. P value less than 0.05 was considered as statistically significant, all *p* values were two sided.

## Results

All one hundred-twenty patients with *H. pylori* infection had positive ^14^C-UBT. Rapid urease test was positive in 116 out of 120 (97%) and negative in 4 out of 120 (3%). Histology demonstrated *H. pylori* associated gastritis in 114 out of 120 (95%) and nonspecific gastritis in 6(5%). Four patients negative by RUT had positive C-14 UBT and *H. pylori* positive gastritis while six patients demonstrating nonspecific gastritis had positive ^14^C-UBT and RUT. The distribution of negative RUT (*p* = 1 and *p* = 0.69) and histology (*p* = 0.600 and *p* = 0.095) for *H. pylori* infection were not associated with age and gender.

### Recurrence of H. pylori after successful eradication

One hundred-twenty patients had positive ^14^C-UBTs on initial visit. After treatment 18 out of 120 (15%) were still positive and were excluded from the study. At 12 months, the ^14^C-UBT confirmed recurrence in six patients. No statistically significant difference was seen for sex (Fisher’s Exact Test *p =* 1) or age (*p =*0.697). All cases of recurrence were from the local population. They were diagnosed as having non-ulcer dyspepsia (NUD) with evidence of endoscopic gastritis and mild to moderate chronic active gastritis on histology (Table 2). In the follow-up period, none of the 3(3%) duodenal ulcer patients with successful eradication had any symptoms suggesting ulcer though we did not do repeat endoscopy in these patients.

**Table 2:** Comparison of histology with diagnosis, bacterial colonization and antibiotic susceptibility

	**Diagnosis**			***H. pylori *DNA fingerprint**		**Clarithromycin**			**Amoxicillin**	
	**Gastritis**	**Gastric Ulcer**	**Duodenal ulcer**	**Same on both sides**	**Different on both sides**	**Sensitive**	**Resistant**	**P**	**Sensitive**	**Resistant**
**Histology**										
Grade 1	60(52)	0(0)	1(33)	48(47)	13(72)	12(40)	11(65)		22(49)	1(50)
Grade 2	51(44)	1(100)	2(67)	49(48)	5(28)	18(60)	5(29)		22(49)	1(50)
Grade 3	5(4)	0(0)	0(0)	5(5)	0(0)	0(0)	1(6)		1(2)	0(0)

*n (%) = number and percentage.

### PCR-based RFLP analysis

In 103 of 120 isolates (86%), we demonstrated the same DNA fingerprint from isolates from the antrum and mid-body gastric sites, and the patterns were different in 17(14%). Six of the 102 patients had recurrence of *H. pylori* during the follow-up period after treatment. Five of these 6 patients were previously documented to have colonization by multiple *H. pylori* isolates while in one patient a single *H. pylori* isolate was present on antrum and corpus. Following recurrence, 4 out of 6 patients had colonization by multiple *H. pylori* isolates and 2 single *H. pylori* isolate on antrum and body.

### Antibiotic susceptibility of H. pylori isolates

Forty-seven *H. pylori* isolates were cultured from pretreatment biopsies. *H. pylori* culture was negative from both antrum and corpus in patient having a positive ^14^C-UBT at 12 month and follow up endoscopic with biopsies. Clarithromycin sensitivity was present in 30 out of 47 (64%) and amoxicillin in 45 out of 47 (98%), respectively (Table 2).

### Comparison of antibiotic susceptibility and H. pylori DNA fingerprint at gastric site

*H. pylori* DNA fingerprints were different in antrum and corpus in 3(10%) out of 30 CLR sensitive strains compared to 10(59%) out of 17 CLR resistance *H. pylori* strains (*p* = 0.001).

By RFLP 18 patients had different DNA fingerprints in the antrum and corpus (Table 2). Of these 18 patients, *H. pylori* could be cultured from only 13 patients. Clarithromycin resistance was demonstrated by 10 out of 13 *H. pylori* isolates while 3 out of 13 were sensitive to CLR.

### Comparison of histology with diagnosis and antibiotic susceptibility

The degree of gastric inflammation in majority of patients varied from grade 1 to 2 (Table 2). It was not significantly associated with diagnosis (*p* = 0.66) (Table 2). Grades of inflammation were equally associated with same or different *H. pylori* DNA fingerprints in antrum and corpus (*p* = 0.08) (Table 2). Eleven out of 17 (65%) *H. pylori* strains demonstrating CLR resistance were associated with grade 1 inflammation (*p* = 0.06) while grade 1 to 2 inflammation were equally associated with AMX resistance (*p* = 0.957), respectively (Table 2).

## Discussion and conclusion

The results of this prospective study showed that rate of *H. pylori* recurrence after apparently successful eradication was low. Majority of our patients remained infection free at nine months and recurrence followed in about 6% at the end of one year. All the patients with recurrence had NUD while none occurred in cases with duodenal ulcer. Clarithromycin resistance was high in our *H. pylori* isolates and low for amoxicillin. Antibiotic susceptibility pattern was not found to be related to age and gender in this study. About 14% of our patients demonstrated colonization by multiple *H. pylori* strains. Clarithromycin resistance was associated with different *H. pylori* strains at different gastric sites as suggested by DNA fingerprinting. However, the technique used in this study looked at only one gene, and generated as few as 2 or 3 bands in some strains, making it a rather low resolution technique to declare a strain identical. Strains may show identical banding patterns in the RFLP sites in one gene, yet differ elsewhere. Furthermore, it is possible that in some subjects, the infection was eradicated, yet the subject was reinfected from family members with an identical or near-identical strain, which would be interpreted as recrudescence.

Most studies from developed countries reported less than 1% rate of *H. pylori* infection recurrence whereas relatively higher rates have been reported from developing countries [18]. True re-infection of *H. pylori* is defined as where tests for *H. pylori* infection stay negative for 12 months after eradication, and become positive again at a later stage. This is probably a rare event in developed countries. A study from the Netherlands [19] showed that at 6 years after successful triple therapy the recurrence rate of *H. pylori* infection was very low (0.19% per patient year). Recurrence rate is higher in developing countries. In a study from Bangladesh, recrudescence, associated with nitroimidazole-based treatment, occurred in 15 of 105 patients (13%) within the first 3 months while the annual reinfection rate was 13%, based on a total follow-up of 84.7 patient years [8]. Data from India on reinfection of *H. pylori* after eradication showed that the risk is low in Indian subjects at the end of one year. The eradication rate with the four-drug regimen was 89.1% (41/46). Four of the 5 non-responders eradicated *H. pylori* with the second regimen. At the end of median one year follow-up (range 9–15 months), one of the 45 patients (2.4%) who eradicated the organism developed reinfection; none of the 46 patients who were initially *H. pylori*-negative acquired new infection [9]. In Iran, 37 patients, aged 5 to 17 years, treated with triple omeprazole based regimen the reinfection rate of *H. pylori* was determined during a follow up period of 12 months. After eradication therapy of *H. pylori,* 34 patients had a negative repeat ^13^C-UBT. Reinfection occurred in 5 (14.7%) patients [10].

The recurrence rate of 6% in our study is similar to that reported from Chile and China (4.2% /yr. and 1.08% /yr., respectively) [20,21]. It is, however, less than that reported in Korea (13%) [22] and Bangladesh (13%). This discrepancy might be explained by the fact that criteria to define eradication of infection, number of patients studied, and time of follow-up varied from one study to another [8,20-22]. Increased susceptibility of hosts to *H. pylori* infection and re-exposure to *H. pylori* are proposed to be the major requirements for re-infection of *H. pylori*[23,24]. Poor sanitation practices in the developing countries result in contamination of the environment with *H. pylori*, such as in drinking water, practice of eating uncooked vegetables; crowded living conditions thatl contribute to re-exposure to *H. pylori* infection and result in high prevalence of *H. pylori* infection [25-27]. Genetic factors may also play a role in re-infection of *H. pylori* infection after successful eradication. Susceptible individuals who have had *H. pylori* eradicated may be prone to re-infection with the bacterium when they are exposed to *H. pylori*-positive persons [28]. Self-prescription is also common in developing countries as medications are sold without prescriptions by pharmacies [29]. Most reports on antimicrobial therapy of *H.pylori* propose that antibiotic overuse selects for resistant strains as they eradicate the susceptible *H. pylori* population, and resistant survivors replace them as a resistant majority [30-33]. The resistance trait could be spread horizontally by plasmids to other bacterial population. Emergence of a resistance phenotype is a short-term phenomenon that takes 4 – 5 years to emerge and the driving force is the indiscriminate, short-interval and frequent use of antibiotics [33-35]. Clarithromycin resistance rate was high in *H. pylori* isolates probably contributed to by use of this drug for other indications in the community and this might result in selection of stable macrolide-resistant *H.pylori* and indigenous microbiota [36]. Also, previously a low cure rate and a higher resistance to clarithromycin were observed among *H. pylori* positive patients with functional dyspepsia than that in peptic ulcer disease [37-39]. In an earlier study, we reported treatment failure was associated with younger mean age, *cagA* negativity and point mutations in *23S rRNA* gene of *H. pylori*[40]. In conclusion, in spite of having a high prevalence of *H. pylori* there is a low incidence of recurrence of *H. pylori* infection in our population once eradication has been achieved. In view of high CLR resistance, judicious use of antibiotics should follow in general.

## Competing interest

The authors declare that they have no competing interests.

## Authors’ contributions

SA and JY conceived and designed the study, JY did the work; SA, JY, ZAB, KM, WJ and SH coordinated the study, RA did the histopathology; JY, SA and ZA analyzed the data, JY performed the statistical analysis. JY wrote the manuscript. All authors read and approved the final manuscript.

## Pre-publication history

The pre-publication history for this paper can be accessed here:

http://www.biomedcentral.com/1471-230X/13/33/prepub
